# Correction: Three-dimensional TiNb_2_O_7_ anchored on carbon nanofiber core–shell arrays as an anode for high-rate lithium ion storage

**DOI:** 10.1039/d4ra90159b

**Published:** 2025-01-23

**Authors:** Meili Qi, Dongliang Chao, Weifeng Sun, Jinghua Yin, Minghua Chen

**Affiliations:** a Key Laboratory of Engineering Dielectric and Applications (Ministry of Education), School of Materials Science and Engineering, Harbin University of Science and Technology Harbin 150080 P. R. China mhchen@hrbust.edu.cn; b School of Materials Science and Engineering, Nanyang Technological University 637553 Singapore

## Abstract

Correction for ‘Three-dimensional TiNb_2_O_7_ anchored on carbon nanofiber core–shell arrays as an anode for high-rate lithium ion storage’ by Meili Qi *et al.*, *RSC Adv.*, 2020, **10**, 6342–6350, https://doi.org/10.1039/C9RA10485B.

The authors regret that there was an error in Fig. 5a.

The *in situ* Raman spectra at 1.25 V during the discharge process for the TNO/CNF core/shell array electrode, as shown in Fig. 5a of the published article, contained an error. Specifically, a duplicated spectrum (the green line in Fig. 5a) was inadvertently included.

The incorrect spectrum (the green line in Fig. 5a) has been replaced with the correct one, and the corrected *in situ* Raman spectra for the TNO/CNF core/shell array electrode during the discharge process are provided here.
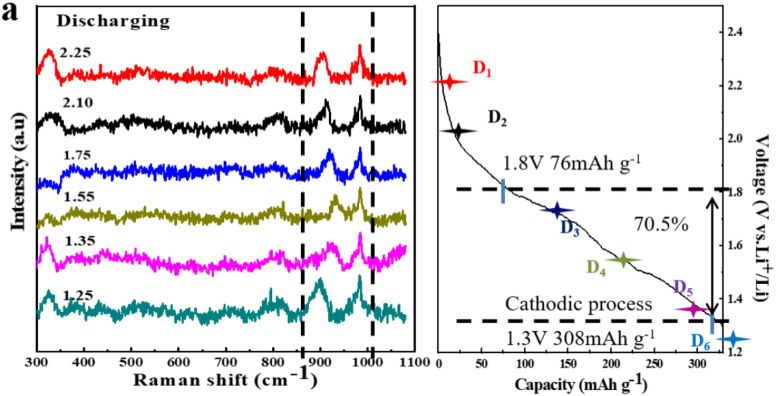


**Fig. 5a** *In situ* Raman spectra evolution of the TNO/CNF core/shell array electrode at different (a) discharge curves of the TNO/CNF electrode.

An independent expert has viewed the corrected figure and has concluded that it is consistent with the discussions and conclusions presented.

The Royal Society of Chemistry apologises for these errors and any consequent inconvenience to authors and readers.

